# Erxian Decoction Attenuates TNF-α Induced Osteoblast Apoptosis by Modulating the Akt/Nrf2/HO-1 Signaling Pathway

**DOI:** 10.3389/fphar.2019.00988

**Published:** 2019-09-10

**Authors:** Nani Wang, Hailiang Xin, Pingcui Xu, Zhongming Yu, Dan Shou

**Affiliations:** ^1^Department of Medicine, Tongde Hospital of Zhejiang Province, Hangzhou, China; ^2^School of Pharmacy, Zhejiang Chinese Medical University, China; ^3^School of Pharmacy, Second Military Medical University, China

**Keywords:** network pharmacology, Erxian decoction, osteoporosis, Akt, tumor necrosis factor

## Abstract

Erxian decoction (EXD), a traditional Chinese medicine formula, has been used for treatment of osteoporosis for many years. The purpose of this study was to investigate the pharmacological effect of EXD in preventing osteoblast apoptosis and the underlying mechanism of prevention. Putative targets of EXD were predicted by network pharmacology, and functional and pathway enrichment analyses were also performed. Evaluations of bone mineral density, serum estradiol level, trabecular area fraction, serum calcium levels, and tumor necrosis factor (TNF)-α levels in ovariectomized rats, as well as cell proliferation assays, apoptosis assays, and western blotting in MC3T3-E1 osteoblasts were performed for further experimental validation. Ninety-three active ingredients in the EXD formula and 259 potential targets were identified. Functional and pathway enrichment analyses indicated that EXD significantly influenced the PI3K-Akt signaling pathway. *In vivo* experiments indicated that EXD treatment attenuated bone loss and decreased TNF-α levels in rats with osteoporosis. *In vitro* experiments showed that EXD treatment increased cell viability markedly and decreased levels of caspase-3 and the rate of apoptosis. It also promoted phosphorylation of Akt, nuclear translocation of transcription factor NF-erythroid 2-related factor (Nrf2), and hemeoxygenase-1 (HO-1) expression in TNF-α-induced MC3T3-E1 cells. Our results suggest that EXD exerted profound anti-osteoporosis effects, at least partially by reducing production of TNF-α and attenuating osteoblast apoptosis *via* Akt/Nrf2/HO-1 signaling pathway.

## Introduction

Osteoporosis is a skeletal disease characterized by imbalanced bone homeostasis, which leads to an increase in bone fragility and fracture risk (Liu et al., 2017). Development of osteoporosis is mainly due to the production of a large number of immune and hematopoietic factors in the bone microenvironment (Yu and Wang, 2016). These complex and interacting factors influence the formation and absorption of bone (Wei and Frenette, 2018). One of the most important factors in osteoporosis is tumor necrosis factor-alpha (TNF-α), which is the strongest bone resorption enhancer and also inhibits bone formation (Zhou et al., 2017a; Zhou et al., 2017b).

Erxian decoction (EXD) is a traditional Chinese medicine (TCM) formulation comprising six herbs: *Epimedium sagittatum* (Siebold & Zucc.) Maxim. (ES), *Curculigo orchioides* Gaertn. (CO), *Angelica sinensis* (Oliv.) Diels. (AS), *Phellodendron chinense* Schneid. (PC), *Anemarrhena asphodeloides* Bge. (AR), and *Morinda officinalis* How (MO). EXD has been used to treat osteoporosis for several decades (Wang et al., 2016). We previously reported that some components of EXD, such as icariin, curculigoside, and berberine, displayed inhibitory effects on osteoclastic bone resorption and positive effects on osteoblast proliferation (Wang et al., 2017a; Wang et al., 2017b). However, potential effects of EXD on TNF-α production and TNF-α-induced bone loss have not been investigated.

Recently, network pharmacology analyses have been used to investigate TCM formulas to predict the molecular targets and pathways of different diseases (Zhao and He, 2018). As a systems biology-based methodology, network pharmacology provides an effective approach for evaluating the multi-pharmacological effects of traditional medicines at the molecular level and for evaluating the interactions of chemical molecules and target proteins (Liu et al., 2016). In our previous study, network pharmacology was used to predict the mechanism for the effects of CO in the prevention and treatment of osteoporosis (Wang et al., 2017a; Wang et al., 2017b). In the current study, network pharmacology was combined with experimental validation to study the effects of EXD on TNF-α-induced bone loss and clarify the underlying mechanism.

## Materials and Methods

### Instruments and Reagents

Double distilled water of at least 18.2 MΩ was purified by an ultrapure water system (Millipore Corporation, Boston, Massachusetts, USA). α-Modified minimum essential medium (α-MEM), phosphate buffered saline (PBS), trypsin, and fetal bovine serum (FBS) were purchased from Gibco (Gaithersburg, Maryland USA). TNF-α (purity >98%) was obtained from Sigma (St Louis, MO, USA).

Orcinol glucosid (>98%), palmatine (>99%), jatrorrhizine (>94%), berberine (>98%), protodioscin (>98%), baohuoside I (>99%), timosaponin BII (>99%), icariin (>98%), obacunone (>8%), curculigoside (>98%), anhydroicaritin (>98%), mangiferin (>98%), epimedin C (>98%), epimedin B (>98%), epimedin A (>98%), magnolflorine (>98%), and phellodendrine (>98%) standards were purchased from Aoke Biological Technology Co., LTD (Beijing, China). Ferulic acid (>98%) and naringin (>98%) were purchased from the National Institutes for Food and Drug Control (Beijing, China). Anemarsaponin B (>98%) was purchased from Yuanye Biological Technology Co. Ltd. (Shanghai, China).

The aerial parts of *E. sagittatum* (Siebold & Zucc.) Maxim. (Lot No: 170420, Drug name: Epimedii Folium) were obtained from Huadong Medicine Co. Ltd. (Zhejiang, China). The rhizomes of *C. orchioides* Gaertn. (Lot No: 1702074, Drug name: Curculiginis Rhizoma), the roots of *M. officinalis* How (Lot No: 1711067, Drug name: Morindae Officinalis Radix), the bark of *P. chinense* Schneid. (Lot No: 1710100, Drug name: Chinensis Cortex), and the rhizomes of *A. asphodeloides* Bge. (Lot No: 1710006, Drug name: Anemarrhenae Rhizoma) were obtained from Quzhou Nankong Chinese Medicine Co. Ltd. (Zhejiang, China). The roots of *A. sinensis* (Oliv.) Diels (Lot No: 1802011, Drug name: Angelicae Sinensis Radix) were obtained from Zhejiang Conba Pharmaceutical Co. Ltd. (Zhejiang, China).

### Chemical Components of Herbs in Erxian Decoction

Chemical components of each herb in EXD were determined from the Traditional Chinese Medicine Systems Pharmacology (TCMSP) (Ru et al., 2014), TCM database @taiwan (Sanderson, 2011), Herbal Ingredients Targets (HIT), Traditional Chinese Medicine Integrated Database (TCMID) (Xue et al., 2013), and previous literature (Bian et al., 2013; Yu et al., 2013). The molecular properties of the herbs, including molecular weight (MW), Moriguchi octanol-water partition coefficient (AlogP), oral bioavailability (OB), drug-likeness (DL), number of donor atoms for H-bonds (nHDon), and number of acceptor atoms for H-bonds (nHAcc) were compared in **Table S1**.

### Predication of Active Components and Targets

OB was used to monitor drug convergence during the ADME process, representing the percentage of an orally administered dose of unchanged drug that reached the systemic circulation (Simpson et al., 2009). DL was used for estimating how “drug-like” prospective compounds were a parameter that is applied in drug design to optimize pharmacokinetic and pharmaceutical properties (Gozalbes et al., 2010). EXD components were considered active if OB ≥30% and DL ≥0.18 (Wang et al., 2015). Validated targets of potential active components were determined from Therapeutic Target Database (Chen et al., 2002), TCMSP, Drug bank (Law et al., 2014), and STITCH (Kuhn et al., 2008).

### Network Pharmacology Analyses

**Figure 1:**
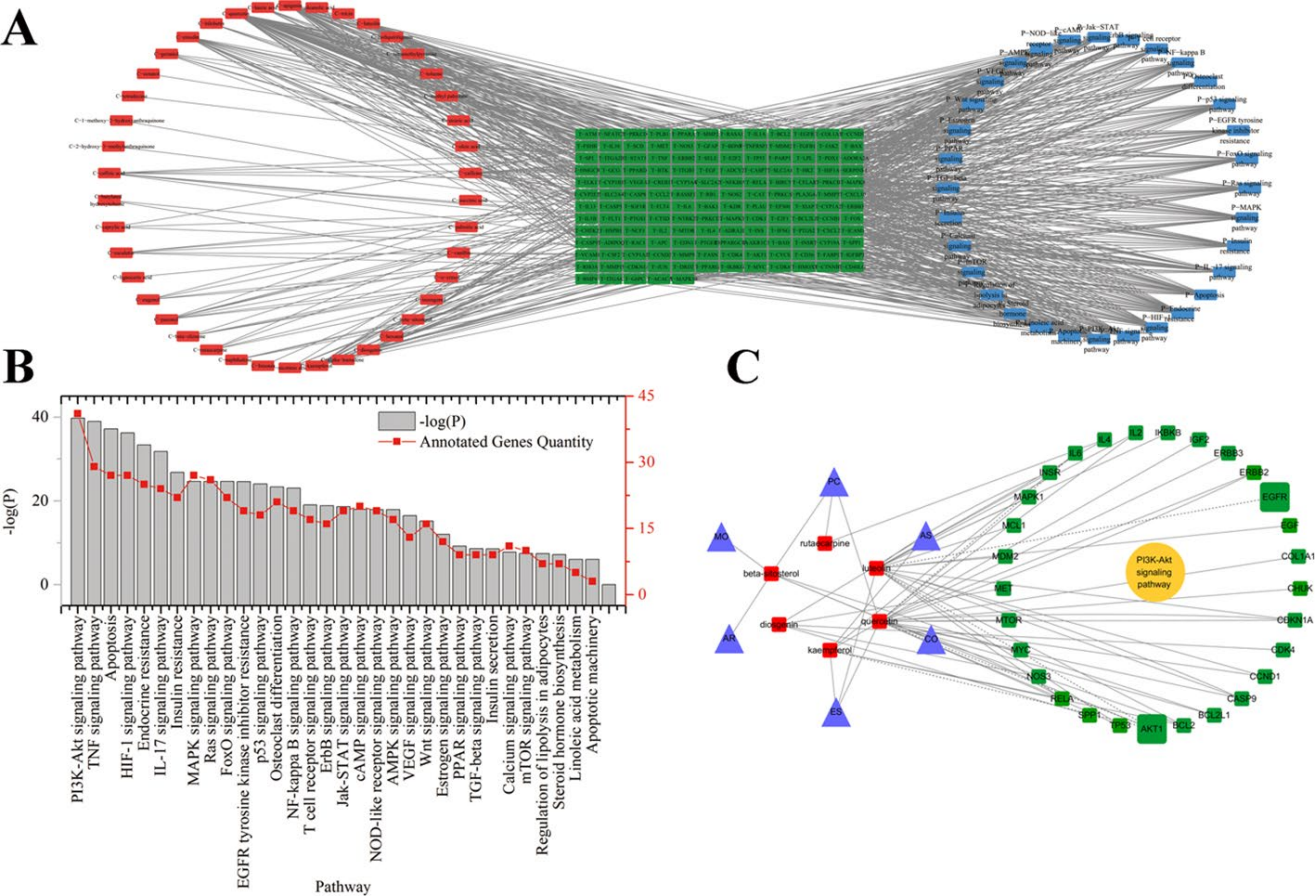
**(A)** Potential active ingredient-target-pathways network of EXD acting on osteoporosis. The network was based on the interaction among ingredients in EXD, active components, and osteoporosis-related targets. Red nodes represented the ingredients; green nodes represented the targets; blue nodes represented the osteoporosis-related pathways. **(B)** KEGG pathways for potential targets of Erxian decoction. **(C)** The interaction among herbs, components, and targets in PI3K-Akt signaling pathway.

Gen Ontology (GO) function enrichment analysis and KEGG (Kyoto Encyclopedia of Genes and Genomes) pathway enrichment analysis were carried out using The Database for Annotation, Visualization and Integrated Discovery (DAVID) database (Huang et al., 2007). The interaction network of the potential active components and identified targets of EXD, as well as of enriched KEGG pathways, were visualized with Cytoscape v3.4.0 software (**Figure 1**).

### Herbal Preparation

EXD extract was prepared by hot water extraction as previously described (Wang et al., 2017a; Wang et al., 2017b) with some modifications. Specifically, herbs were pulverized and 20 g of the powder of each herb (120 g total) was placed in 5-L triangular flasks. Samples were extracted twice with 2-L water (1 h each extraction) at 100°C. Extracted solutions were concentrated to 1 g (crude extract)/ml.

### Animals and Treatment

Twelve-month-old female Wistar rats (weighting 350–400 g) were supplied by the Animal Center of the Zhejiang Academy of Traditional Chinese Medicine (Hangzhou, China). The rats were maintained in air-conditioned quarters at 24 ± 2°C and a relative humidity of 60 ± 5%. All protocols for animal experiments were approved in accordance with the Guide for the Care and Use of Laboratory Animals and were approved by the Bioethics Committee of Zhejiang Academy of Traditional Chinese Medicine.

Animals were body-weight matched and randomly assigned into several groups: 1) sham-operated group (Control group); 2) ovariectomy (OVX) group (Model group); and 3) three OVX operated groups with oral administration of EXD extract (EXD groups). The doses of EXD administered were 2, 4, and 6 g/kg/day in the low, middle, and high level to EXD groups, respectively. EXD administration was initiated 4 days after OVX operation and provided for 12 weeks.

### Liquid Chromatographic Analysis

High-performance liquid chromatography (HPLC) was performed on a Thermo Fisher ultra-high-performance liquid chromatography 3000 system (Thermo Fisher Technologies, Waltham, MA, USA), comprising a dual pump, auto-sampler, ultraviolet (UV) detector and Chromeleon software equipped with a 4.6 × 250 mm ZORBAX Eclipse XDB C18 column (Agilent, Santa Clara, CA, USA). The column temperature was set at 30°C. The mobile phases of HPLC were composed of acetonitrile (A) and water (B). The analytical column was SHISEIDO Capcell Pak C18 (Tokyo, Japan). The gradient condition was as follows: 0–15 min, 95–80% B; 15–30 min, 80–60% B; 30–60 min, 60–40% B. The column flow rate was 1.00 ml/min. The wavelength of UV detection was set at 285 nm. The injection volume was 10 μl. The EXD extract was air-dried and the residue was dissolved to a final concentration of 5 mg/ml in a 1:9 mixture of acetonitrile and water. The diluted solution was passed through a 0.22-µM pore filter prior to HPLC analysis.

### Histomorphology Assay

Left femurs were fixed in 10% formalin, embedded in paraffin, cut into 5-μm-thick sections, and finally stained with hematoxylin–eosin (HE) for histopathological analysis. At least 10 independent fields were assessed per sample in each treatment group. Trabecular area was measured according to the previous literature (Kloefkorn and Allen, 2016). Bone mineral density (BMD) was determined by Lunar dual-energy X-ray absorptiometry (GE, Boston, MA, USA) using the small animal scan mode.

### Serum Biochemical Analysis

Blood was drawn from rats and centrifuged at 4°C, 5,000 × g for 10 min to collect the upper serum, and stored at −80°C. The concentrations of serum calcium (Ca), estradiol (E_2_), TNF-α, and caspase-3 were detected with commercial detection kits (Boster Biological Technology Co. Ltd, California, USA) in accordance with the manufacturers’ protocols.

### Immunohistochemistry Analysis

Osteocalcin expression in the right femur was examined by immunohistochemistry. Briefly, tissue sections were treated with 3% H_2_O_2_ to remove endogenous peroxidase, incubated with anti-osteocalcin antibody (ab13420, Abcam, Cambridge, UK) at 4°C overnight prior to addition of added secondary antibody (PV-6002, ZSGB-Bio, Beijing, China), incubated at 37° C for 30 min, then colored with DAB (diaminobenzidine) reaction staining. Finally, sections were evaluated by microscopy in a blinded manner.

MT3T3-E1 (5 × 10^6^) cells were seeded in six-well plates and then treated with EXD extract or TNF-α as indicated. Cells were fixed with 4% paraformaldehyde in PBS (0.1 M, pH 7.4) for 15 min, permeabilized with 50 μg/ml digitonin in PBS for 5 min, blocked with 0.1% (v/v) gelatin in PBS for 30 min, and then incubated with primary antibodies for 1 h. After washing, cells were incubated with Alexa Fluor 488-conjugated goat anti-guinea pig and Alexa Fluor 647-conjugated goat anti-rabbit IgG secondary antibodies (Invitrogen, Waltham, MA, USA) for 30 min. Cells were imaged using a laser-scanning microscope (LSM510 META, Carl Zeiss, Oberkochen, Germany) with a Plan Apochromat 63 × NA 1.4 oil differential interference contrast objective lens.

### Cell Culture and Treatment

MC3T3-E1 cells were cultured in α-MEM containing 10% FBS. All cells were washed with PBS before incubation with EXD or TNF-α. After reaching 85% confluence, the cells were treated with medium containing TNF-α and EXD according to the experimental design.

### Cell Proliferation Assay

Cell viability was evaluated with commercially available 3-(4,5-dimethylthiazol-2-yl)-2,5-diphenyltetrazolium bromide (MTT) detection kits (Bio-Rad, Foster, California, USA) in accordance with the manufacturer’s protocol. Briefly, treated cells were incubated 10% MTT solution at 37°C for 4 h, supernatants were removed, and the formazan crystals were dissolved in 150 μl DMSO (dimethyl sulfoxide). Absorbance was recoded at a wavelength of 490 nm.

### Assay of Annexin V-EGFP/PI Apoptosis

Apoptosis was determined using the Annexin V-EGFP/PI Apoptosis Detection Kit (Jiancheng, Nanjing, China). MT3T3-E1 cells (5 × 10^6^) were seeded in six-well plates and then treated with LY294002, EXD extract, or TNF-α according to the experimental design. Detection was performed using flow cytometric analysis (ACCURI C6, BD Bioscience, Franklin Lakes, NJ, USA).

### Western Blot Analysis

MT3T3-E1 cells (5 × 10^6^) were seeded in six-well plates and then treated with EXD extract or TNF-α according to the experimental design. Cells were extracted with lysis buffer for 30 min at 4°C and the supernatant containing total protein was harvested. Aliquots containing 50 μg of protein were separated by sodium dodecylsulfate polyacrylamide gel electrophoresis and transferred to a polyvinylidine difluoride membrane (Millipore, MA, USA). Membranes were soaked in blocking buffer (5% skimmed milk) for 2 h. The proteins were detected with primary antibodies overnight at 4°C, and then probed with HRP (horseradish peroxidase)-conjugated secondary antibody for 1 h at room temperature. Detection was performed with an enhanced chemiluminescence system (ECL, Beyotime, Haimen, China). The relative content of each protein of interest was calculated as the ratio of its optical density to that of β-actin in the same sample.

### Statistical Analysis

All experiments were performed in triplicate and results are presented as mean ± SD. One-way ANOVA was used to assess statistical significance of differences between group means. A value of p < 0.05 was considered statistically significant.

## Results

### Physicochemical Properties of Active Components in EXD

A total of 93 compounds present in EXD met the criteria for inclusion (OB ≥ 30% and DL index ≥ 0.18). MW, AlogP, HDon, nHAcc, OB, and DL of all 93 compounds were determined to identify similarities and differences in the physicochemical properties of the components in each herb (**Table 1**). Herbal sources of these EXD components were determined from TCMSP, TCM database @taiwan, HIT, TCMID, and previous reports, as shown in **Table S1**. The structures of these potentially active components are presented in **Table S2**. The nHacc and DL of the components derived from ES were significantly different to those derived from PC, and the nHacc of compounds derived from AR were different from those from all other herbs.

**Table 1:** Properties of potential active ingredients from Erxian decoction.

Herb^a^	MW	AlogP	Hdon	Hacc	OB (%)	DL
ES	355.95 ± 111.69	3.36 ± 2.13^*^	2.77 ± 2.15^*^	5.27 ± 3.67^**^	48.36 ± 15.83	0.42 ± 0.23
CO	438.09 ± 184.08	4.77 ± 2.94	3.10 ± 2.88	4.80 ± 4.81^**^	46.01 ± 15.47	0.41 ± 0.29
MO	329.16 ± 88.24	3.51 ± 3.57	1.79 ± 1.45^*^	4.66 ± 3.07^**^	57.51 ± 28.23	0.36 ± 0.25
AS	407.69 ± 141.08	3.76 ± 2.05	2.53 ± 2.09^*^	4.95 ± 3.59^*^	51.66 ± 24.96	0.52 ± 0.28
PC	371.18 ± 97.28	4.16 ± 2.09	1.07 ± 1.47^##^	4.29 ± 2.03^**^	42.24 ± 10.80	0.60 ± 0.25^##^
AR	303.35 ± 94.34	5.71 ± 1.77^#^	0.40 ± 0.49^#^	0.40 ± 0.49^##^	52.37 ± 11.13	0.38 ± 0.31

Epimedium sagittatum (Siebold & Zucc.) Maxim. (ES), Curculigo orchioides Gaertn. (CO), Angelica sinensis (Oliv.) Diels. (AS), Phellodendron chinense Schneid. (PC), Anemarrhena asphodeloides Bge. (AR), Morinda officinalis How (MO).

^#^*P* < 0.05, ^##^ *P* < 0.01 compared to ES; ^*^P < 0.05, ^**^P < 0.01 compared to AR.

### Network Pharmacology Analysis of EXD

A total of 259 targets corresponding to active components in EXD were collected. Cytoscape was used to establish the chemical component-targets-pathway regulatory network of EXD, which showed correlation of 42 compounds, 150 targets, and 32 pathways, as shown in **Figure 1**. The network among PI3K-Akt pathway, the targets, and ingredients were presented in **Figure 1** **C**. According to the degree ranking in the network, the top 10 nodes were AKT1 (degree 108), TP53 (degree 102), IL6 (degree 99), VEGFA (degree 95), CASP3 (degree 89), JUN (degree 88), PTGS2 (degree 86), MAPK8 (degree 85), MAPK1 (degree 83), and EGFR (degree 80). AKT1 appeared with the highest frequency and was correlated with 132 nodes, including 5 components, 19 KEGG pathways, and 108 targets. Among the 42 ingredients, luteolin from ES had the highest degree (degree 31). PI3K-Akt had the highest degree (40) in the signaling pathway. Six EXD components would have a close relationship with Akt signaling pathway. The network analysis suggested that the Akt pathway may play an important role in the anti-osteoporosis mechanism of EXD.

### EXD Attenuated Osteoporosis in Ovariectomy Rats

**Figure 2:**
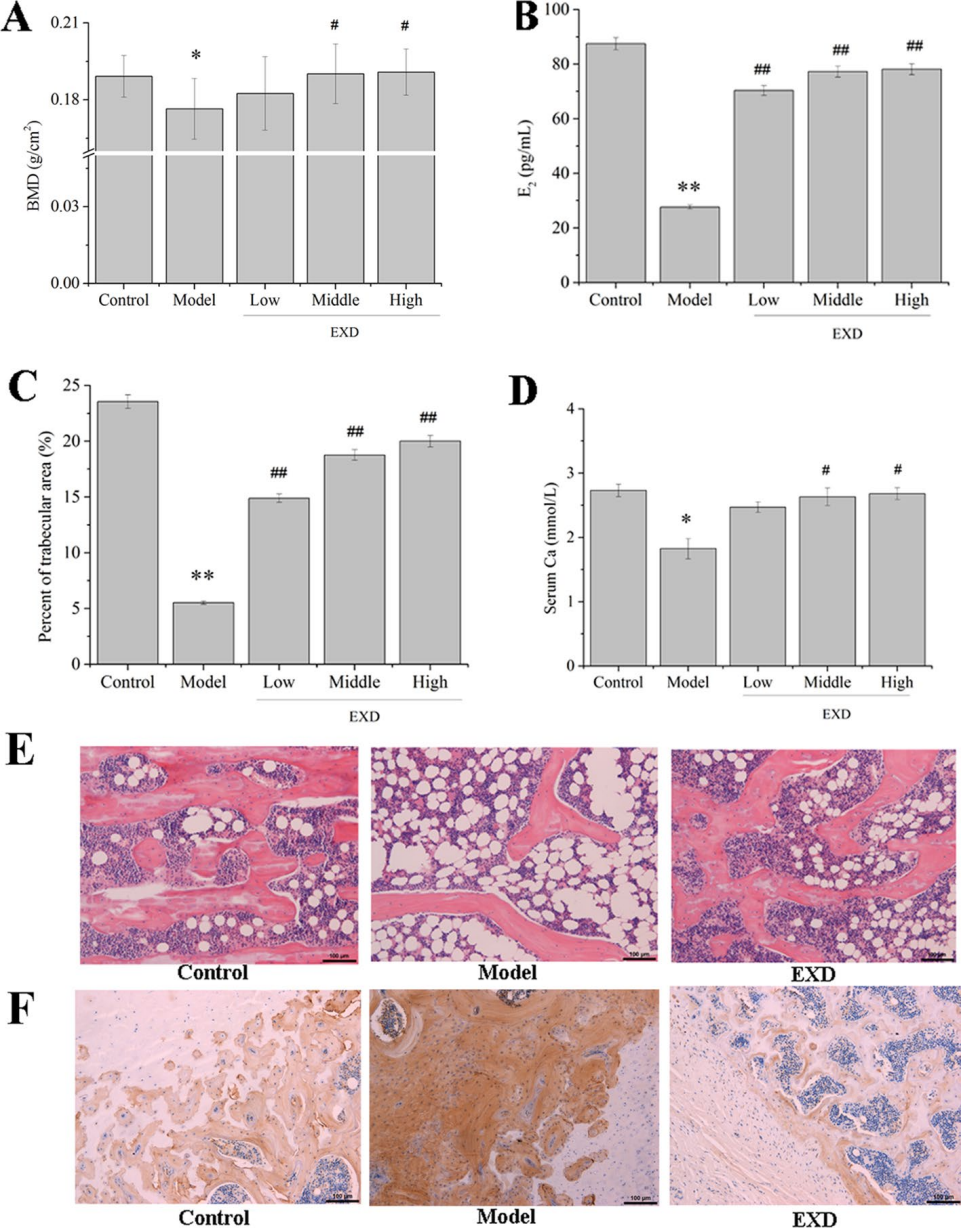
EXD ameliorated osteoporosis in OVX rats. Wistar rats were administrated with water or EXD extract at a dose of 2 g/kg/day (low level), 4 g/kg/day (middle level), 6 g/kg/day (high level), which started on day 4 after OVX operation for 12 weeks. **(A)** The bone mineral density. **(B)** Serum estradiol level. **(C)** Percent of trabecular area. **(D)** Serum Ca level. **(E)** Representative images of femur stained with hematoxylin–eosin (magnification, ×200). Oral administration dose of EXD was 4 g/kg/day. **(F)** Representative images of femur stained with osteocalcin antibody (magnification, ×200). Oral administration dose of EXD was 4 g/kg/day. ^#^*P* < 0.05, ^##^*P* < 0.01 compared to the control group. ^*^P < 0.05, ^**^P < 0.01 compared to the model group.

Twenty dominant components in the EXD extract were quantified by HPLC-UV (**Figure S1**). These components ranged in concentration from 0.23 to 34.38 mg/L (**Table S3**), with icariin from ES, orcinol glucoside from CO, and epimedin B from ES being the most abundant compounds. As compared with the model group, BMD was significantly higher in the EXD group (**Figure 2A**). EXD treatment also increased the fraction of trabecular area of OVX rats, E_2_ level, serum Ca, and bone formation (**Figures 2B–E**). As shown in **Figure 2F**, osteocalcin was negatively correlated with BMD, trabecular area, and E_2_ level. It is possible that a high transformation type of osteoporosis was induced by OVX resulting in bone absorption being higher than bone formation (Zhou et al., 2017a; Zhou et al., 2017b). After EXD treatment, osteocalcin expression markedly reduced.

**Figure 3:**
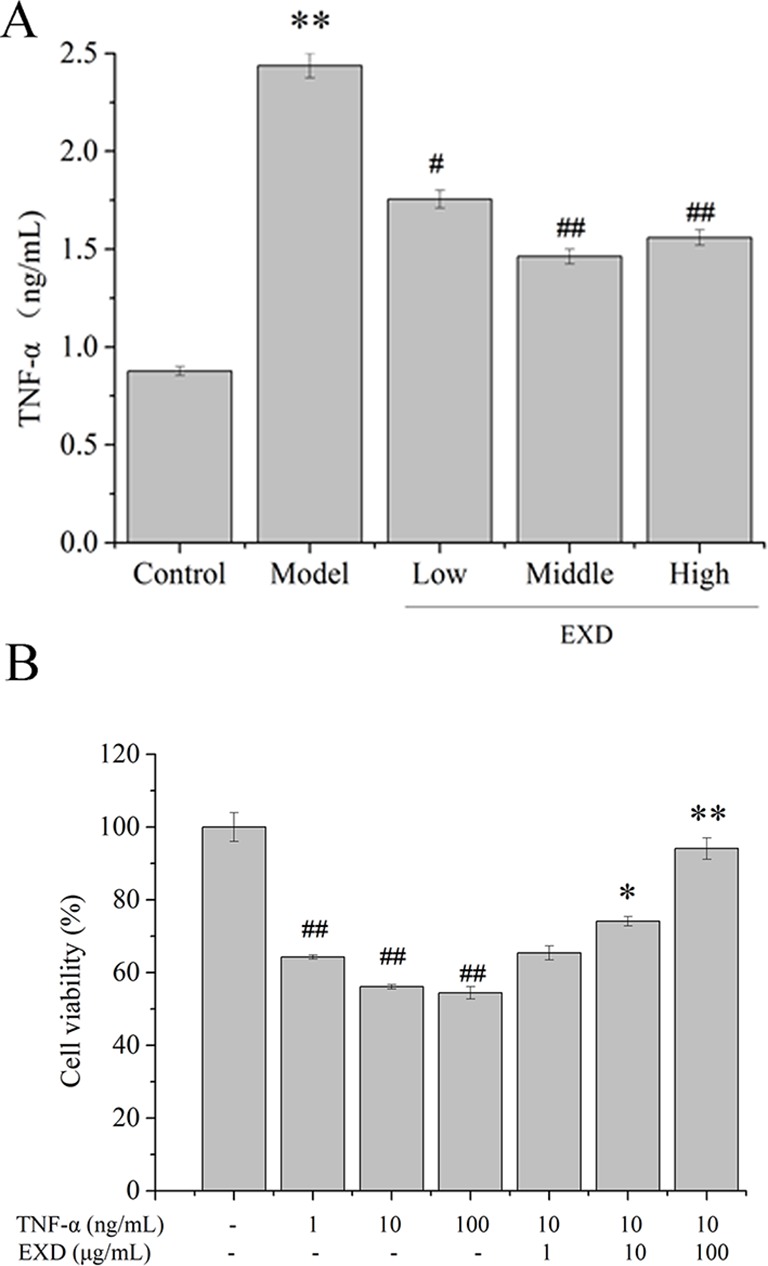
**(A)** TNF-α level in ovariectomy rats. Wistar rats were administrated with water or EXD extract at a dose of 2 g/kg/day (low level), 4 g/kg/day (middle level), 6 g/kg/day (high level), which started on day 4 after OVX operation for 12 weeks. ^#^ *P* <0.05, ^##^ *P* < 0.01 compared to the control group. ^*^P < 0.05, ^**^P < 0.01 compared to the model group. **(B)** Effects of EXD on MC3T3-E1cell viability. Cells were induced by TNF-α. ^#^*P* <0.05, ^##^*P* < 0.01 compared to the control group. ^*^P < 0.05, ^**^P < 0.01 compared to the TNF-α group (10 ng/ml).

The level of TNF-α in the serum of the model group was 170% higher than in the control group (**Figure 3**). Compared with the model group, the EXD groups had an average 44% decrease in the TNF-α level.

### EXD Protects Osteoblasts Against TNF-α Induced Injury Through AKT/Nrf2/HO-1 Pathway

To investigate the potential effects of EXD on TNF-α-induced cytotoxicity in osteoblasts, MC3T3-E1 osteoblastic cells were treated with TNF-α or EXD in a range of concentrations (0.1–10 μM). Cell viability decreased after exposure to TNF-α and EXD treatment significantly protected MC3T3-E1 cell against TNF-α-induced injury in a dose-dependent manner (**Figure 3**).

**Figure 4:**
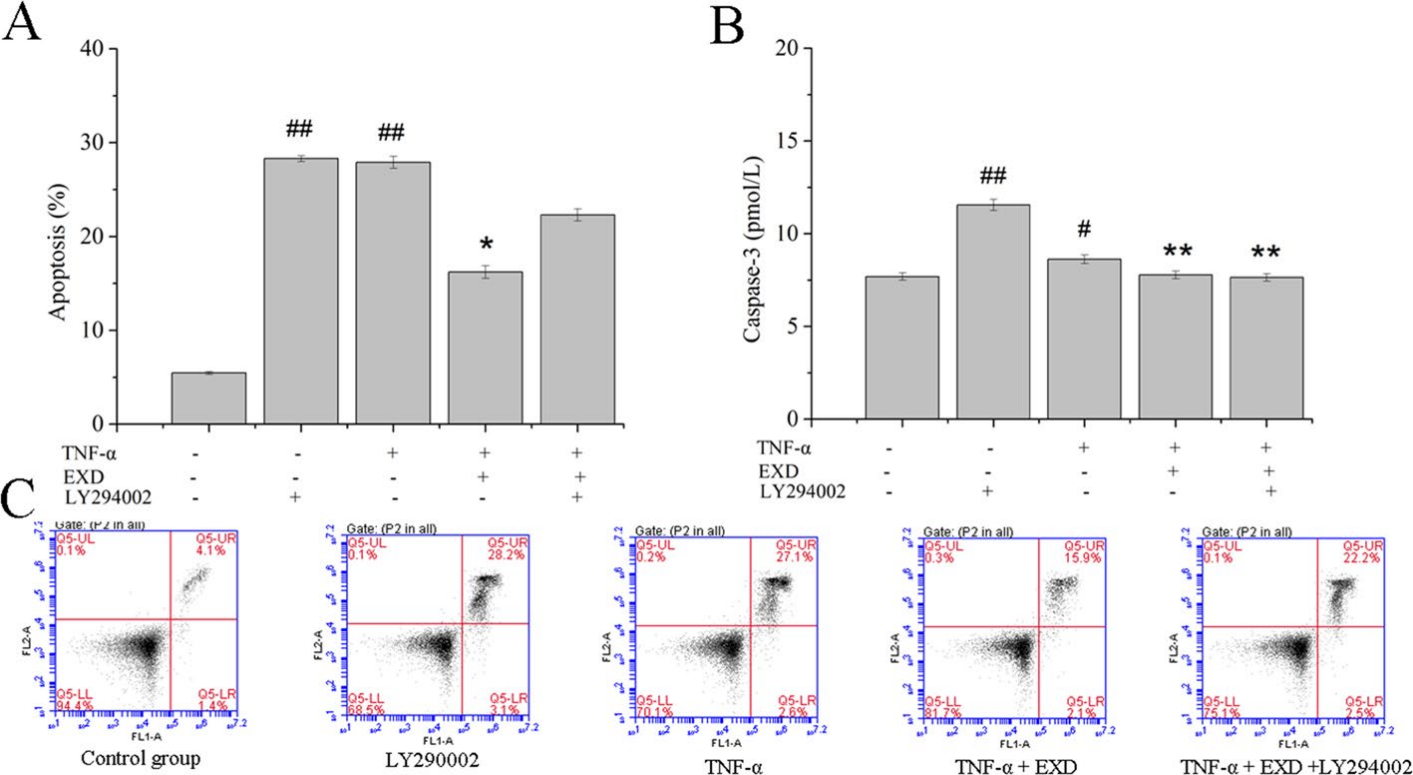
Effect of EXD and selective inhibitor LY294002 on TNF-α-induced apoptosis in MC3T3-E1 cells. Cells were incubated with or without 20 μM LY294002, 100 μg/ml EXD, and 10 ng/ml TNF-α for 24 h. **(A)** Cell apoptosis rate. **(B)** Caspase-3 level. **(C)** Annexin V/PI staining results. ^#^*P* < 0.05, ^##^*P* < 0.01 compared to the control group. ^*^P < 0.05, ^**^P < 0.01 compared to the TNF-α group.

**Figure 5:**
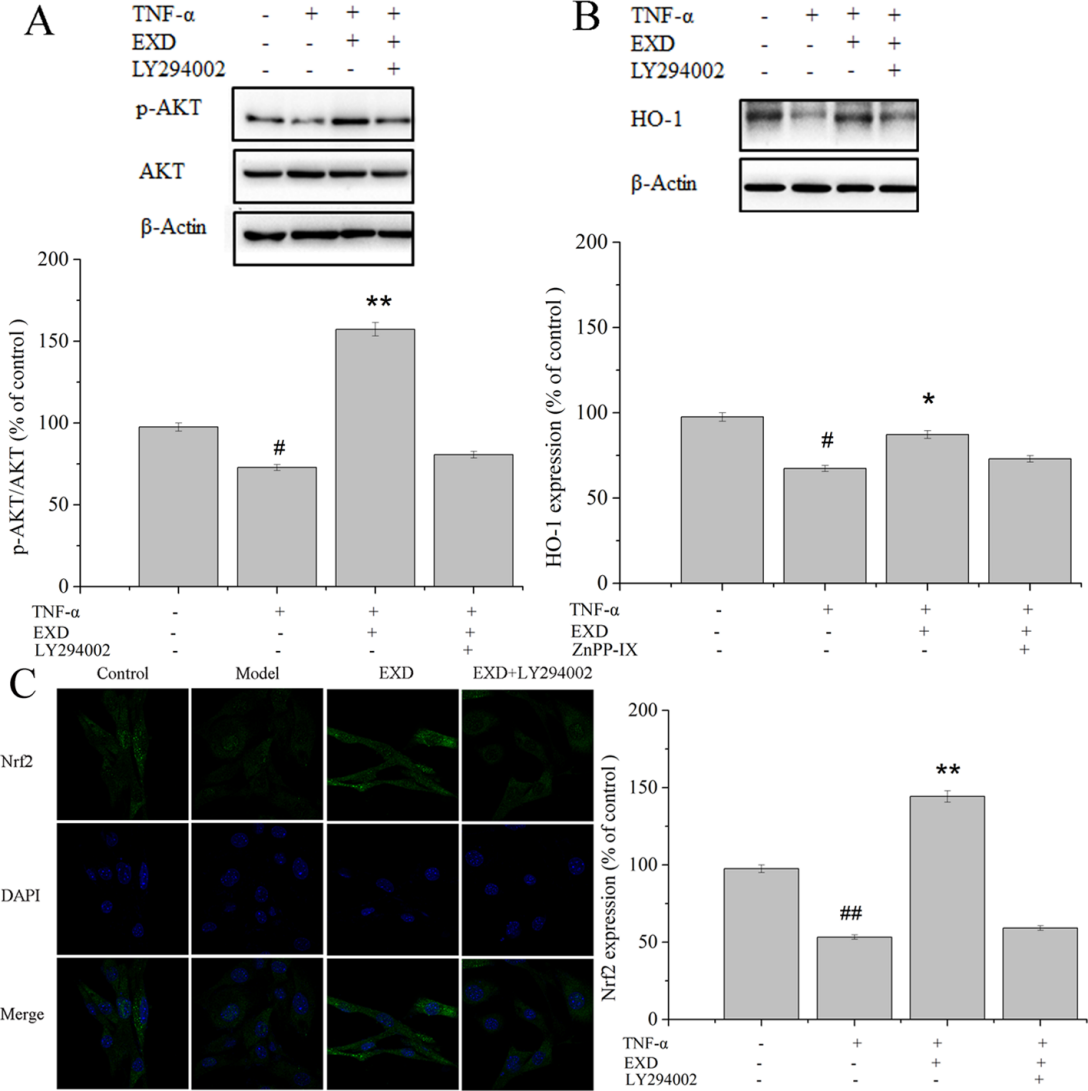
Effect of EXD and selective inhibitor LY294002 on **(A)** AKT, **(B)** HO-1, and **(C)** Nrf2 expression (magnification, ×650). Cells were incubated with or without 20 μM LY294002, 5 μM ZnPP-IX, 100 μg/ml EXD, and 10 ng/ml TNF-α for 24 h. ^#^*P <* 0.05, ^##^*P* < 0.01 compared to the control group. ^*^P < 0.05, ^**^P < 0.01 compared to the TNF-α group.

EXD treatment reduced apoptosis after TNF-α exposure (**Figure 4**) and increased phosphorylation of AKT. It also significantly activated the expression of nuclear Nrf2 and HO-1, which were the essential downstream targets of Akt (**Figure 5**). PI3K inhibitor (LY294002) reduced both p-Akt and nuclear Nrf2 expression. A HO-1 inhibitor (ZnPP-IX) reduced the expression of HO-1 in the EXD-treated osteoblasts. Taken together, the above results indicated that EXD reduced TNF-α production and cell apoptosis, probably *via* activation of the Akt/Nrf2/HO-1 signaling pathway in TNF-α induced osteoblast.

## Discussion

**Figure 6:**
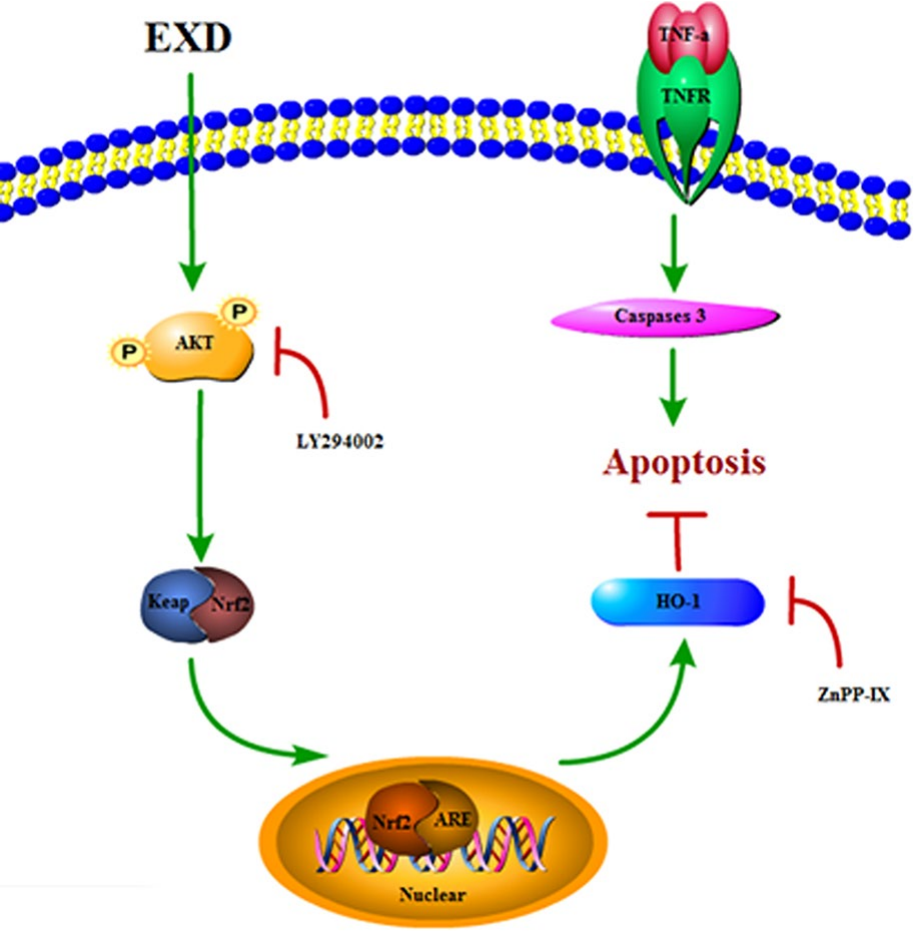
A proposed signaling pathway involved in EXD against TNF-α-induced cell damage.

EXD, a traditional Chinese medicinal formula, has been used widely for treating osteoporosis (Tong et al., 2012). However, the effect of EXD on osteoblast injury induced by TNF-α induced underlying mechanism has not been fully identified. In the current study, EXD treatment attenuated TNF-α production in OVX rat and protected osteoblasts against TNF-α-induced apoptosis. Our results suggest that EXD might attenuate osteoporosis at least partially by regulating the Akt/Nrf2/HO-1 signaling pathway (**Figure 6**).

*In vivo* investigation showed significantly decreased TNF-α serum levels after EXD treatment. Similarly, we showed that TNF-α reduced osteoblast activity and ALP activity *in vitro*, resulting in increased apoptosis and activation of caspase-3. The cleavage of caspase-3 is the final step of the process that initiates the apoptotic signaling (Wei and Frenette, 2018). However, EXD attenuated the increase of caspase-3 and TNF-α compared to the cell model. Thus, EXD treatment not only attenuated TNF-α production in the serum, but also protected osteoblasts against TNF-α-induced apoptosis.

Molecular networks were constructed to demonstrate interactions between EXD ingredients, targets, and enriched pathways. Pathway and functional enrichment analyses indicate that EXD was primarily associated with the PI3K-Akt signaling pathway, which in turn was associated with 29 targets (**Figure 1**). Investigation of the correlation between PI3K-Akt activation and EXD-regulated osteoblast protection in the presence of TNF-α found results consistent with results from the network pharmacology study, i.e., EXD administration activated the PI3K-Akt pathway. A PI3K inhibitor (LY294002) reduced EXD-mediated protection of cell death and apoptosis (**Figure 4**), in accordance with the previous reports that activation of PI3K-Akt pathway attenuates osteoblast injury in osteoporosis (Vanella et al., 2010).

An increasing number of studies have indicated that TNF-α exposure significantly effects HO-1 expression, which is an essential anti-inflammatory molecule that regulates pro-inflammatory mediators (Chiu et al., 2016). Since the master upstream regulator of HO-1, Nrf2, is affected by the PI3K-Akt signaling pathway, we examined whether the Akt/Nrf2/HO-1 signaling pathway was involved in EXD mediated regulation of osteoblast apoptosis. EXD was found to increase the level of AKT phosphorylation and also promoted nuclear translocation of Nrf2, further increasing HO-1 expression. LY294002 inhibited nuclear translocation of Nrf2, and a HO-1 inhibitor (ZnPP-IX) reversed the EXD-mediated increase of HO-1 expression in TNF-α-induced osteoblasts. These observations suggest that EXD protected osteoblasts against TNF-α-induced apoptosis at least partially by activating the Akt/Nrf2/HO-1 signaling pathway.

Furthermore, the molecular network analysis predicted that six EXD components would have a close relationship with Akt signaling pathway, including kaempferol, luteolin, quercetin, beta-sitosterol, diosgenin, and rutaecarpine. The above six EXD compounds were derived from the traditional medicinal parts of six herbs in EXD. Three flavonoids (luteolin, kaempferol, and quercetin) could be isolated from the aerial parts of *E. sagittatum* (Siebold & Zucc.) Maxim. (Chen et al., 1996; Wang et al., 2010a; Wang et al., 2010b). Diosgenin was a spirostanol steroidal saponin, which could be obtained from the rhizomes of *A. asphodeloides* Bge. (Xia et al., 2017). These ingredients showed anti-osteoporosis effects *via* promotion of osteogenic differentiation and stimulation of mineralization in osteoblasts (Pang et al., 2006; Nash et al., 2015). Luteolin and kaempferol also had anti-apoptotic properties (Pang et al., 2006; Im et al., 2018). Beta-sitosterol existed in four herbs, including bark of *P. chinense* Schneid (Wang et al., 2009; Xue et al., 2015), roots of *M. officinalis* How. (Hong et al., 2009; Zhang et al., 2018), rhizomes of *A. asphodeloides* Bge. (Bian et al., 1996), and rhizomes of *C. orchioides* Gaertn. (Cao et al., 2009). Beta-sitosterol could promote the proliferation and mineralized nodule formation of osteoblasts (Chauhan et al., 2018). Rutaecarpine could be found in the fruit and bark of *P. chinense* Schneid. (Yan et al., 2017). Future investigations will evaluate the relationship between the above ingredients and the Akt/Nrf2/HO-1 signaling pathway.

## Conclusions

In the current study, we showed that EXD protects osteoblasts against apoptosis following exposure to excess TNF-α and used network modeling to elucidate the underlying mechanism of this protection. EXD ameliorated osteoporosis and reduced TNF-α level in OVX rats in the dose range of 2–6 g/kg/day. Further studies will be carried out in order to explore the potential of EXD at a lower concentration range. Combining network pharmacology analysis with experimental verification *in vivo* and *in vitro*, we found that EXD may attenuate osteoporosis at least partially by reducing TNF-α production and regulating the Akt/Nrf2/HO-1 signaling pathway.

## Ethics Statement

Twelve-month-old female Wistar rats (weighting 350-400 g) were supplied by the Animal Center of the Zhejiang Academy of Traditional Chinese Medicine (Hangzhou, China). The rats were maintained in air-conditional animal quarters at a temperature of 24 ± 2°C and a relative humidity of 60 ± 5%. All protocols for animal experiments were approved in accordance with the regulations of experimental animal administration issued by the state commission of science and technology of the People’s Republic of China.

## Author Contributions

NW designed and performed experimental procedures, including network pharmacology analysis and *in vitro* experiments. PX developed the osteoporosis rat models. HX helped with *in vivo* experiments and provided professional consultancy about pharmaceutical use. ZY assisted in preparing samples and provided medical materials. DS revised the manuscript critically for important intellectual content.

## Conflict of Interest Statement

The authors declare that the research was conducted in the absence of any commercial or financial relationships that could be construed as a potential conflict of interest.

## Supplementary Material

Figure S1 **(A)** Typical chromatograms of the EXD extract and the standards. **(B)** The chemical structure of the peaks. Other information of the peaks was presented in **Table S3**.
