# Supplementary figures and images for: Erxian Decoction Attenuates TNF-α Induced Osteoblast Apoptosis by Modulating the Akt/Nrf2/HO-1 Signaling Pathway
Source: Front Pharmacol. 2019 Sep 10;10:988. doi: 10.3389/fphar.2019.00988 (PMC6748068; doi:10.3389/fphar.2019.00988)

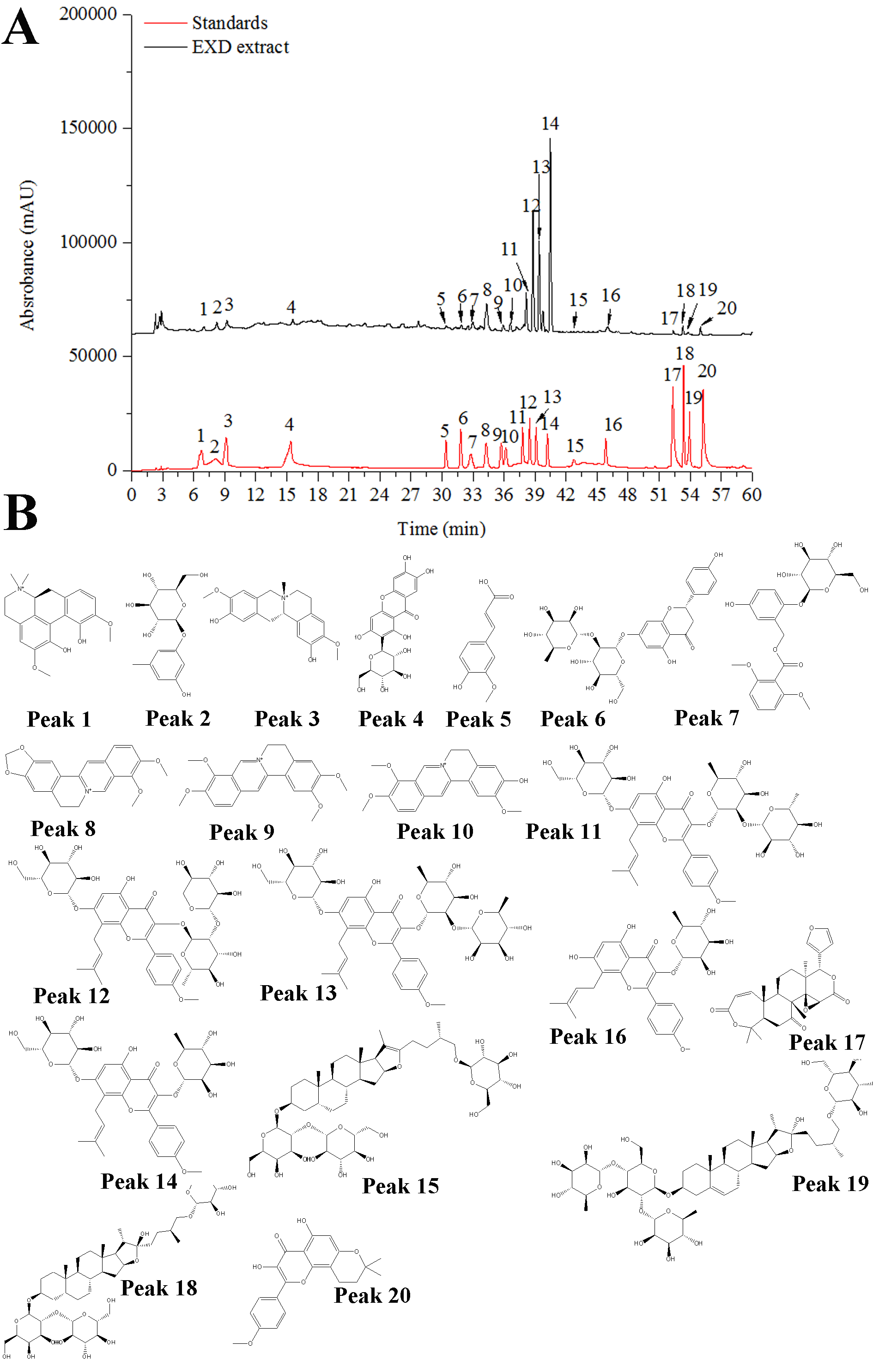

Supplement: Figure S1 — (A) Typical chromatograms of the EXD extract and the standards. (B) The chemical structure of the peaks. Other information of the peaks was presented in Table S3 . [file Image_1.tif]
