# Supplementary material for: Erxian Decoction Attenuates TNF-α Induced Osteoblast Apoptosis by Modulating the Akt/Nrf2/HO-1 Signaling Pathway
Source: Front Pharmacol. 2019 Sep 10;10:988. doi: 10.3389/fphar.2019.00988 (PMC6748068; doi:10.3389/fphar.2019.00988)
Supplement: Supplementary file 2 [file Table_1.docx]

Table S1 Potential active ingredients in EXD

| Herb source | Molecule Name | MW | AlogP | Hdon | Hacc | OB (%) | Caco-2 | BBB | DL | FASA- | HL |
| --- | --- | --- | --- | --- | --- | --- | --- | --- | --- | --- | --- |
| ES | 1,2-Bis(4-hydroxy-3-methoxyphenyl)propan-1,3-diol | 320.37 | 1.69 | 4 | 6 | 52.31 | 0 | -0.87 | 0.22 | 0 | 2.18 |
| ES | Icariin | 676.73 | 0.77 | 8 | 15 | 41.58 | -1.82 | -3 | 0.61 | 0 | 19.93 |
| ES | Icariin I | 676.73 | 0.77 | 8 | 15 | 41.58 | -2.06 | -3.06 | 0.61 | 0.25 | 18.02 |
| ES | Icariside A7 | 462.49 | 1.16 | 5 | 10 | 31.91 | -0.65 | -1.56 | 0.86 | 0 | 2.84 |
| ES | Linoleyl acetate | 308.56 | 6.85 | 0 | 2 | 42.1 | 1.36 | 1.08 | 0.2 | 0.21 | 7.48 |
| ES | Luteolin | 286.25 | 2.07 | 4 | 6 | 36.16 | 0.19 | -0.84 | 0.25 | 0.39 | 15.94 |
| ES | Olivil | 376.44 | 1.68 | 4 | 7 | 62.23 | -0.16 | -0.75 | 0.41 | 0 | 2.27 |
| ES | Yinyanghuo A | 420.49 | 4.2 | 3 | 6 | 56.96 | 0.38 | -0.49 | 0.77 | 0 | 14.44 |
| ES | Yinyanghuo C | 336.36 | 3.39 | 2 | 5 | 45.67 | 0.75 | -0.11 | 0.5 | 0 | 15.74 |
| ES | Yinyanghuo E | 352.36 | 3.12 | 3 | 6 | 51.63 | 0.51 | -0.54 | 0.55 | 0 | 15.47 |
| ES | 24-Epicampesterol | 400.76 | 7.63 | 1 | 1 | 37.58 | 1.43 | 1.15 | 0.71 | 0.21 | 4.5 |
| ES | 6-Hydroxy-11,12-dimethoxy-2,2-dimethyl-1,8-dioxo-2,3,4,8-tetrahydro-1H-isochromeno[3,4-h]isoquinolin-2-ium | 370.41 | 2.75 | 1 | 6 | 60.64 | 0.34 | -0.12 | 0.66 | 0 | 1.22 |
| ES | 8-(3-Methylbut-2-enyl)-2-phenyl-chromone | 290.38 | 4.99 | 0 | 2 | 48.54 | 1.53 | 0.99 | 0.25 | 0 | 18.73 |
| ES | 8-Isopentenyl-kaempferol | 354.38 | 3.63 | 4 | 6 | 38.04 | 0.53 | -0.49 | 0.39 | 0 | 15.37 |
| ES | Anhydroicaritin-3-O-alpha-L-rhamnoside | 676.73 | 0.77 | 8 | 15 | 41.58 | -1.59 | -2.89 | 0.61 | 0 | 16.23 |
| ES | C-Homoerythrinan, 1,6-didehydro-3,15,16-trimethoxy-, (3.beta.)- | 329.48 | 2.89 | 0 | 4 | 39.14 | 1.02 | 0.68 | 0.49 | 0 | 6.58 |
| ES | Chryseriol | 300.28 | 2.32 | 3 | 6 | 35.85 | 0.39 | -0.53 | 0.27 | 0.32 | 16.31 |
| ES | DFV | 256.27 | 2.57 | 2 | 4 | 32.76 | 0.51 | -0.29 | 0.18 | 0.42 | 17.89 |
| ES, PC | Magnograndiolide | 266.37 | 1.18 | 2 | 4 | 63.71 | 0.02 | -0.24 | 0.19 | 0.3 | 3.17 |
| ES, PC | Poriferast-5-en-3beta-ol | 414.79 | 8.08 | 1 | 1 | 36.91 | 1.45 | 1.14 | 0.75 | 0 | 5.07 |
| ES, PC | Quercetin | 302.25 | 1.5 | 5 | 7 | 46.43 | 0.05 | -0.77 | 0.28 | 0.38 | 14.4 |
| ES, PC | Sitosterol | 414.79 | 8.08 | 1 | 1 | 36.91 | 1.32 | 0.87 | 0.75 | 0.22 | 5.37 |
| ES | Anhydroicaritin | 368.41 | 3.88 | 3 | 6 | 45.41 | 0.72 | 0.01 | 0.44 | 0 | 15.01 |
| PC | (S)-Canadine | 339.42 | 3.4 | 0 | 5 | 53.83 | 1.01 | 0.64 | 0.77 | 0.19 | 6.56 |
| PC | Berberine | 336.39 | 3.45 | 0 | 4 | 36.86 | 1.24 | 0.57 | 0.78 | 0.19 | 6.57 |
| PC | Berberrubine | 322.36 | 3.2 | 1 | 4 | 35.74 | 1.07 | 0.17 | 0.73 | 0.24 | 6.46 |
| PC | Campesterol | 400.76 | 7.63 | 1 | 1 | 37.58 | 1.34 | 0.95 | 0.71 | 0.22 | 4.83 |
| PC | Candletoxin A | 608.79 | 3.63 | 2 | 9 | 31.81 | -0.39 | -1.24 | 0.69 | 0.32 | -0.36 |
| PC | Cavidine | 353.45 | 3.72 | 0 | 5 | 35.64 | 1.08 | 0.63 | 0.81 | 0 | 5.78 |
| PC | Chelerythrine | 332.37 | 4.29 | 0 | 4 | 34.18 | 1.24 | 0.28 | 0.78 | 0.36 | 6.32 |
| PC | Coptisine | 320.34 | 3.25 | 0 | 4 | 30.67 | 1.21 | 0.32 | 0.86 | 0.26 | 9.33 |
| PC | Dehydrotanshinone II A | 292.35 | 4.22 | 0 | 3 | 43.76 | 1.02 | 0.52 | 0.4 | 0.33 | 23.71 |
| PC | Delta 7-stigmastenol | 414.79 | 8.08 | 1 | 1 | 37.42 | 1.3 | 0.83 | 0.75 | 0.22 | 5.27 |
| PC | Delta7-Dehydrosophoramine | 242.35 | 1.09 | 0 | 3 | 54.45 | 0.99 | 0.75 | 0.25 | 0.22 | 5.52 |
| PC | Dihydroniloticin | 458.8 | 5.66 | 2 | 3 | 36.43 | 0.65 | 0.03 | 0.81 | 0.25 | 7.04 |
| PC | Fumarine | 353.4 | 2.95 | 0 | 6 | 59.26 | 0.56 | -0.13 | 0.83 | 0.3 | 23.46 |
| PC | Hericenone H | 580.88 | 9.95 | 0 | 6 | 39 | 0.8 | 0.16 | 0.63 | 0.22 | 3.62 |
| PC | Hispidone | 472.78 | 4.46 | 2 | 4 | 36.18 | 0.12 | -0.72 | 0.83 | 0.25 | 5.08 |
| PC | Isocorypalmine | 341.44 | 3.35 | 1 | 5 | 35.77 | 0.85 | 0.43 | 0.59 | 0.21 | 2.47 |
| PC | Kihadalactone A | 512.7 | 3.96 | 0 | 7 | 34.21 | 0.19 | -0.51 | 0.82 | 0.29 | -6.27 |
| PC | Kihadanin A | 486.56 | 1.76 | 1 | 9 | 31.6 | -0.93 | -1.36 | 0.7 | 0.38 | 2.68 |
| PC | Melianone | 470.76 | 4.76 | 1 | 4 | 40.53 | 0.4 | -0.31 | 0.78 | 0.27 | 6.09 |
| PC | Niloticin | 456.78 | 5.62 | 1 | 3 | 41.41 | 0.54 | -0.2 | 0.82 | 0.27 | 5.19 |
| PC | N-Methylflindersine | 241.31 | 1.72 | 0 | 3 | 32.36 | 1.35 | 1.1 | 0.18 | 0.33 | 0.41 |
| PC | Obacunone | 454.56 | 2.68 | 0 | 7 | 43.29 | 0.01 | -0.43 | 0.77 | 0.31 | -13.04 |
| PC | Palmatine | 352.44 | 3.65 | 0 | 4 | 64.6 | 1.33 | 0.37 | 0.65 | 0.13 | 2.25 |
| PC | Palmidin A | 510.52 | 4.52 | 6 | 8 | 35.36 | -0.38 | -1.47 | 0.65 | 0.39 | 33.17 |
| PC | Phellamurin_qt | 356.4 | 3.61 | 4 | 6 | 56.6 | 0.14 | -0.5 | 0.39 | 0.38 | 14.89 |
| PC | Phellavin_qt | 374.42 | 2.51 | 5 | 7 | 35.86 | -0.41 | -1.32 | 0.44 | 0.33 | 15.81 |
| PC | Phellochin | 488.83 | 5.26 | 2 | 4 | 35.41 | 0.47 | -0.01 | 0.82 | 0.24 | 6.64 |
| PC | Phellopterin | 300.33 | 3.64 | 0 | 5 | 40.19 | 0.98 | 0.48 | 0.28 | 0.24 | -1.64 |
| PC | Rutaecarpine | 287.34 | 3.36 | 1 | 3 | 40.3 | 1.13 | 0.71 | 0.6 | 0.33 | 8.21 |
| PC | Skimmianin | 259.28 | 2.33 | 0 | 5 | 40.14 | 1.26 | 1.1 | 0.2 | 0.14 | -2.43 |
| PC | Thalifendine | 322.36 | 3.2 | 1 | 4 | 44.41 | 1.12 | 0.21 | 0.73 | 0.22 | 5.99 |
| PC | Worenine | 334.37 | 3.73 | 0 | 4 | 45.83 | 1.22 | 0.24 | 0.87 | 0.27 | 8.41 |
| AA, CO, PC, MO | Beta-sitosterol | 414.79 | 8.08 | 1 | 1 | 36.91 | 1.32 | 0.99 | 0.75 | 0.23 | 5.36 |
| CO | 3,2',4',6'-Tetrahydroxy-4,3'-dimethoxy chalcone | 332.33 | 2.6 | 4 | 7 | 52.69 | 0.45 | -0.15 | 0.28 | 0.26 | 17.94 |
| CO | Curculigosaponin C | 769.09 | 1.2 | 8 | 13 | 39.31 | -2.05 | -3.24 | 0.19 | 0.22 | 11.84 |
| CO | Curculigoside B_qt | 290.29 | 2.45 | 3 | 6 | 83.36 | 0.24 | -0.2 | 0.19 | 0.38 | 5.01 |
| CO | Cycloartenol | 426.8 | 7.55 | 1 | 1 | 38.69 | 1.53 | 1.33 | 0.78 | 0 | 5 |
| CO | ZINC03982454 | 414.79 | 8.08 | 1 | 1 | 36.91 | 1.27 | 0.88 | 0.76 | 0.22 | 3.87 |
| AS | (Z)-3-(4-Hydroxy-3-methoxy-phenyl)-N-[2-(4-hydroxyphenyl)ethyl]acrylamide | 313.38 | 2.86 | 3 | 5 | 118.35 | 0.51 | -0.27 | 0.26 | 0 | 4.26 |
| AS | Anemarsaponin C_qt | 416.71 | 4.97 | 2 | 3 | 35.5 | 0.43 | -0.46 | 0.87 | 0.2 | 3.98 |
| AS | Anemarsaponin E_qt | 448.76 | 4.53 | 2 | 4 | 30.67 | 0.34 | -0.23 | 0.86 | 0.17 | 5.14 |
| AS | Anemarsaponin F_qt | 432.71 | 3.92 | 2 | 4 | 60.06 | 0.43 | -0.33 | 0.79 | 0.18 | 4.61 |
| AS | Asperglaucide | 444.57 | 4.02 | 2 | 6 | 58.02 | 0.28 | -0.22 | 0.52 | 0.42 | 6.88 |
| AS | Chrysanthemaxanthin | 584.96 | 8.24 | 2 | 3 | 38.72 | 0.51 | -0.98 | 0.58 | 0.3 | 17.47 |
| AS | Coumaroyltyramine | 283.35 | 2.88 | 3 | 4 | 112.9 | 0.6 | -0.22 | 0.2 | 0.41 | 5.63 |
| AS | Diosgenin | 414.69 | 4.63 | 1 | 3 | 80.88 | 0.82 | 0.27 | 0.81 | 0.19 | 4.14 |
| AS | Hippeastrine | 315.35 | 1.17 | 1 | 6 | 51.65 | 0.02 | -0.49 | 0.62 | 0.32 | 8.09 |
| AS | Mangiferolic acid | 442.75 | 6.47 | 2 | 3 | 36.16 | 0.66 | -0.06 | 0.84 | 0.25 | 5.71 |
| AS | Marmesin | 246.28 | 2.03 | 1 | 4 | 50.28 | 0.52 | 0.07 | 0.18 | 0.3 | -0.09 |
| AS | Timosaponin B III_qt | 416.71 | 4.77 | 2 | 3 | 35.26 | 0.42 | -0.47 | 0.87 | 0.2 | 4.32 |
| AS, ES | Kaempferol | 286.25 | 1.77 | 4 | 6 | 41.88 | 0.26 | -0.55 | 0.24 | 0 | 14.74 |
| AS, AA, CO, PC | Stigmasterol | 412.77 | 7.64 | 1 | 1 | 43.83 | 1.44 | 1 | 0.76 | 0.22 | 5.57 |
| MO | (2R,3S)-(+)-3',5-Dihydroxy-4 ,7-dimethoxydihydroflavonol | 332.33 | 1.99 | 3 | 7 | 77.24 | 0.13 | -0.39 | 0.33 | 0.29 | 14.13 |
| MO | 1,5,7-Trihydroxy-6-methoxy-2-methoxymethylanthracenequinone | 330.31 | 1.79 | 3 | 7 | 80.42 | 0.27 | -0.63 | 0.38 | 0.24 | 28.25 |
| MO | 1,6-Dihydroxy-5-methoxy-2-(methoxymethyl)-9,10-anthraquinone | 314.31 | 2.06 | 2 | 6 | 104.54 | 0.37 | -0.3 | 0.34 | 0.28 | 29.02 |
| MO | 1-Hydroxy-3-methoxy-9,10-anthraquinone | 254.25 | 2.53 | 1 | 4 | 104.33 | 0.59 | -0.24 | 0.21 | 0.39 | 30.78 |
| MO | 1-Hydroxy-6-hydroxymethylanthracenequinone | 254.25 | 1.94 | 2 | 4 | 81.77 | -0.04 | -0.79 | 0.21 | 0.42 | 30.8 |
| MO | 2-Hydroxy-1,5-dimethoxy-6-(methoxymethyl)-9,10-anthraquinone | 328.34 | 2.31 | 1 | 6 | 95.85 | 0.54 | -0.17 | 0.37 | 0.24 | 29.98 |
| MO | 2-Hydroxy-1,8-dimethoxy-7-methoxymethylanthracenequinone | 328.34 | 2.31 | 1 | 6 | 112.3 | 0.46 | -0.3 | 0.37 | 0.25 | 29.44 |
| MO | 2-Hydroxyethyl 5-hydroxy-2-(2-hydroxybenzoyl)-4-(hydroxymethyl)benzoate | 332.33 | 1.41 | 4 | 7 | 62.32 | -1.02 | -1.71 | 0.26 | 0.37 | 17.61 |
| MO | 3Beta,20(R),5-alkenyl-stigmastol | 414.79 | 8.08 | 1 | 1 | 36.91 | 1.36 | 0.89 | 0.75 | 0.22 | 5.22 |
| MO | 3Beta-24S(R)-butyl-5-alkenyl-cholestol | 456.88 | 9.25 | 1 | 1 | 35.35 | 1.36 | 0.79 | 0.82 | 0.23 | 7.04 |
| MO | Alizarin-2-methylether | 254.25 | 2.53 | 1 | 4 | 32.81 | 0.62 | -0.14 | 0.21 | 0 | 30.82 |
| MO | Americanin A | 328.34 | 2.3 | 3 | 6 | 46.71 | -0.08 | -0.98 | 0.35 | 0.41 | 2.6 |
| MO | Asperuloside tetraacetate | 582.56 | -1.03 | 0 | 15 | 45.47 | -1.39 | -1.73 | 0.82 | 0.34 | 20.72 |
| MO | Diop | 390.62 | 7.44 | 0 | 4 | 43.59 | 0.79 | 0.26 | 0.39 | 0.28 | 3.6 |
| MO | Ethyl oleate (NF) | 310.58 | 7.44 | 0 | 2 | 32.4 | 1.4 | 1.1 | 0.19 | 0.19 | 4.85 |
| MO | Isoprincepin | 494.53 | 2.52 | 5 | 9 | 49.12 | -0.18 | -1.47 | 0.77 | 0.32 | 3 |
| MO | Ohioensin-A | 372.39 | 3.57 | 3 | 5 | 38.13 | 0.6 | -0.17 | 0.76 | 0.41 | 15.09 |
| MO | Supraene | 410.8 | 11.33 | 0 | 0 | 33.55 | 2.08 | 1.73 | 0.42 | 0.27 | 2.72 |
