# Supplementary material for: Erxian Decoction Attenuates TNF-α Induced Osteoblast Apoptosis by Modulating the Akt/Nrf2/HO-1 Signaling Pathway
Source: Front Pharmacol. 2019 Sep 10;10:988. doi: 10.3389/fphar.2019.00988 (PMC6748068; doi:10.3389/fphar.2019.00988)
Supplement: Supplementary file 3 [file Table_2.docx]

Table S2 Structures of the potential active components in EXD

| Name | Structure | Name | Structure |
| --- | --- | --- | --- |
| asperglaucide |  | berberine |  |
| Stigmasterol |  | dihydroniloticin |  |
| Icariin I |  | kihadanin A |  |
| Anemarsaponin C_qt |  | niloticin |  |
| Anemarsaponin E_qt |  | rutaecarpine |  |
| (Z)-3-(4-hydroxy-3-methoxy-phenyl)-N-[2-(4-hydroxyphenyl)ethyl]acrylamide |  | Skimmianin |  |
| diosgenin |  | Chelerythrine |  |
| coumaroyltyramine |  | Stigmasterol |  |
| Marmesin |  | Worenine |  |
| Mangiferolic acid |  | coptisine |  |
| kaempferol |  | Cavidine |  |
| Anhydroicaritin |  | Candletoxin A |  |
| Anemarsaponin F_qt |  | Hericenone H |  |
| Chrysanthemaxanthin |  | Hispidone |  |
| Hippeastrine |  | beta-sitosterol |  |
| Timosaponin B III_qt |  | Magnograndiolide |  |
| beta-sitosterol |  | Palmidin A |  |
| Stigmasterol |  | palmatine |  |
| ZINC03982454 |  | Fumarine |  |
| Cycloartenol |  | Isocorypalmine |  |
| beta-sitosterol |  | Kihadalactone A |  |
| 3,2',4',6'-Tetrahydroxy-4,3'-dimethoxy chalcone |  | quercetin |  |
| Curculigoside B_qt |  | phellamurin_qt |  |
| curculigosaponin C |  | (S)-Canadine |  |
| Stigmasterol |  | poriferast-5-en-3beta-ol |  |
| 24-epicampesterol |  | N-Methylflindersine |  |
| kaempferol |  | berberrubine |  |
| olivil |  | campesterol |  |
| Anhydroicaritin |  | delta7-Dehydrosophoramine |  |
| C-Homoerythrinan, 1,6-didehydro-3,15,16-trimethoxy-, (3.beta.)- |  | Obacunone |  |
| Yinyanghuo A |  | melianone |  |
| Yinyanghuo C |  | phellochin |  |
| Yinyanghuo E |  | thalifendine |  |
| 6-hydroxy-11,12-dimethoxy-2,2-dimethyl-1,8-dioxo-2,3,4,8-tetrahydro-1H-isochromeno[3,4-h]isoquinolin-2-ium |  | Phellavin_qt |  |
| 8-(3-methylbut-2-enyl)-2-phenyl-chromone |  | delta 7-stigmastenol |  |
| Anhydroicaritin-3-O-alpha-L-rhamnoside |  | Phellopterin |  |
| Linoleyl acetate |  | Dehydrotanshinone II A |  |
| 1,2-bis(4-hydroxy-3-methoxyphenyl)propan-1,3-diol |  | Alizarin-2-methylether |  |
| Icariin |  | 2-hydroxy-1,5-dimethoxy-6-(methoxymethyl)-9,10-anthraquinone |  |
| Icariside A7 |  | 1,5,7-trihydroxy-6-methoxy-2-methoxymethylanthracenequinone |  |
| luteolin |  | 1,6-dihydroxy-5-methoxy-2-(methoxymethyl)-9,10-anthraquinone |  |
| Magnograndiolide |  | 1-hydroxy-3-methoxy-9,10-anthraquinone |  |
| quercetin |  | 1-hydroxy-6-hydroxymethylanthracenequinone |  |
| poriferast-5-en-3beta-ol |  | 2-hydroxy-1,8-dimethoxy-7-methoxymethylanthracenequinone |  |
| DFV |  | (2R,3S)-(+)-3',5-Dihydroxy-4 ,7-dimethoxydihydroflavonol |  |
| Chryseriol |  | Supraene |  |
| 8-Isopentenyl-kaempferol |  | 3beta,20(R),5-alkenyl-stigmastol |  |
| sitosterol |  | 3beta-24S(R)-butyl-5-alkenyl-cholestol |  |
| Diop |  | americanin A |  |
| Ethyl oleate (NF) |  | Asperuloside tetraacetate |  |
| beta-sitosterol |  | isoprincepin |  |
| sitosterol |  | 2-hydroxyethyl 5-hydroxy-2-(2-hydroxybenzoyl)-4-(hydroxymethyl)benzoate |  |
| Ohioensin-A |  |  |  |
