# Supplementary material for: Erxian Decoction Attenuates TNF-α Induced Osteoblast Apoptosis by Modulating the Akt/Nrf2/HO-1 Signaling Pathway
Source: Front Pharmacol. 2019 Sep 10;10:988. doi: 10.3389/fphar.2019.00988 (PMC6748068; doi:10.3389/fphar.2019.00988)
Supplement: Supplementary file 4 [file Table_3.docx]

Table S3 Retention time, ratio, and contents of 20 dominating components in EXD extract

| Peak No. | Compound | Retention time  (min) | Ratio^a^  (%) | Content^b^  (mg/L) |
| --- | --- | --- | --- | --- |
| 1 | Magnolflorine | 6.76 | 1.56 | 0.40 |
| 2 | Orcinol glucoside | 8.62 | 2.07 | 25.52 |
| 3 | Phellodendrine | 9.20 | 1.81 | 5.68 |
| 4 | Mangiferin | 15.51 | 1.78 | 5.87 |
| 5 | Ferulic acid | 30.35 | 0.87 | 1.74 |
| 6 | Naringin | 31.85 | 1.44 | 0.24 |
| 7 | Curculigoside | 32.98 | 0.88 | 4.57 |
| 8 | Berberine | 34.87 | 13.09 | 6.85 |
| 9 | Palmatine | 35.9 | 0.21 | 0.23 |
| 10 | Jatrorrhizine | 36.08 | 0.22 | 0.78 |
| 11 | Epimedin A | 38.2 | 5.90 | 7.91 |
| 12 | Epimedin B | 39.04 | 13.62 | 14.80 |
| 13 | Epimedin C | 39.87 | 10.65 | 13.49 |
| 14 | Icariin | 40.85 | 20.96 | 34.38 |
| 15 | Anemarsaponin B | 42.77 | 0.50 | 13.94 |
| 16 | Baohuoside I | 45.82 | 2.05 | 3.82 |
| 17 | Obacunon | 52.23 | 1.70 | 7.15 |
| 18 | Timosaponin BII | 53.14 | 2.29 | 14.61 |
| 19 | Protodioscin | 53.97 | 0.73 | 2.35 |
| 20 | Anhydroicaritin | 55.09 | 2.07 | 3.67 |

^a^ Ratio=Peak area of the analyte/total peak area of EXD × 100%

^b^ The content of the analyte in 5 mg(crude drug)/mL EXD extract.
